# A comparison of pre and post-operative vedaprofen with ketoprofen for pain control in dogs

**DOI:** 10.1186/s12917-015-0338-4

**Published:** 2015-02-07

**Authors:** Denise Tabacchi Fantoni, Keila Kazue Ida, Thais Ingles de Almeida, Aline Magalhães Ambrósio

**Affiliations:** Laboratório de Investigação Médica 8, Anestesiologia, Faculdade de Medicina, Universidade de São Paulo, São Paulo, Brazil, Av. Doutor Arnaldo, 455, 2° andar, sala 2120, Cerqueira César, 01246-903 São Paulo, SP Brazil; Departamento de Cirurgia, Faculdade de Medicina Veterinária e Zootecnia, Universidade de São Paulo, Brazil, Avenida Prof. Orlando Marques Paiva, 87 Cidade Universitária, 05508-270 São Paulo, SP Brazil

**Keywords:** Non-steroidal anti-inflammatory drug, Sublingual, Analgesia, Preventive, Preemptive

## Abstract

**Background:**

This prospective randomized blinded clinical study aimed to investigate the potential of vedaprofen for preventive analgesia, comparing its analgesic effects with ketoprofen administered post-operatively in dogs undergoing maxillectomy or mandibulectomy.

**Results:**

Pain control was effective and rescue analgesia was not necessary in any group. Pain scores were not significantly different between groups. The respiratory rate and rectal temperature were decreased in all groups at extubation until 6 hours post-extubation compared to baseline. Cortisol and epinephrine levels were increased only at 0.5 hours after extubation in all groups compared to baseline.

**Conclusions:**

Vedaprofen did not present any preventive analgesic effect. Pre- and postoperative vedaprofen were as effective as ketoprofen for postoperative pain control.

**Electronic supplementary material:**

The online version of this article (doi:10.1186/s12917-015-0338-4) contains supplementary material, which is available to authorized users.

## Background

Acute pain treated inappropriately after surgery can be magnified and has the potential to develop into a long-term postoperative pain as a result of central sensitization, which is much more difficult to manage [1,2]. Preventive analgesia consists of all perioperative efforts to decrease pain and minimize consumption of analgesics in the postoperative period. This seems to be the most effective means of decreasing postoperative pain, because surgical incision alone is not the only trigger for central sensitization. Other factors, such as preoperative pain and additional painful noxious intraoperative inputs such as retraction, as well as postoperative inflammatory processes, can all result in the development of an altered central processing of afferent input, which, in turn, is capable of amplifying subsequent postoperative nociceptive input [3]. The emphasis of preventive analgesia is on the pathophysiologic phenomenon that should prevent altered sensory processing, and thus investigation of a preventive analgesic effect requires comparison of two groups of patients receiving identical treatments that had been started before and after surgery, respectively [1].

The concept of preventive analgesia was founded on experimental studies of pre-emptive analgesia in animal models of pain [4,5]. Pre-emptive analgesia is an intervention given before incision or surgery that it is more effective than the same treatment administered after incision or surgery. There are only a few investigations fundamentally designed for comparing the pre- versus postoperative administration in veterinary patients [6,7]. In humans, the pre-emptive epidural analgesia with opioids or local anesthetics induces consistent postoperative improvements, and the pre-emptive infiltration of wounds with local anesthetic improves analgesic consumption and time for the first rescue analgesic request [1]. Non-steroidal anti-inflammatory drugs (NSAIDs) also can improve some aspects of postoperative pain when given before surgery compared to the administration after surgery in humans [8-11] and dogs [6,7]. The NSAIDs inhibit peripheral and central prostaglandin synthesis, which reduces the inflammation that accompanies tissue injury, in addition to the attenuation of the response to noxious stimuli [12].

Vedaprofen is a NSAID that can control pain and inflammation associated with clinical and experimental musculoskeletal disorders in dogs [13-15]. It is available as a palatable gel formulation, which has a rapid and almost complete absorption after oral administration in dogs. The oral route has a terminal half-live of the same order of magnitude of the intravenous administration [16], but with the advantage that the oral route is less stressful to the animal and can be easily administered by the owner during postoperative home care [17]. Vedaprofen is known to preferentially inhibit the cyclo-oxygenase-2 in dogs [18], and it is apparently safe in this species, as there was a low incidence of gastrointestinal changes, like vomiting and diarrhea after a long-term treatment for 30–56 days [13,14].

Although vedaprofen has proved to be an effective analgesic for postoperative pain control in dogs, no studies have investigated if the sublingual preoperative administration has a superior analgesic effect over the administration after surgery on postoperative pain control in this species. Therefore, the aim of this prospective, randomized, double-blind study, was to investigate whether sublingual vedaprofen given before was more effective than that given after surgery for decreasing postoperative pain intensity and analgesic consumption in dogs undergoing maxillectomy and mandibulectomy. Since the analgesic effect of sublingual vedaprofen was never assessed in dogs undergoing maxillectomy and mandibulectomy, we included a control group consisting of animals receiving ketoprofen, a NSAID known to provide adequate postoperative analgesia for this surgical procedure in this species [19]. Our hypothesis was that the preoperative administration of vedaprofen would reduce postoperative pain and analgesic requirements more than would vedaprofen or ketoprofen given after surgery.

## Results

There were no significant differences among groups in terms of the study population. No significant differences in the duration of surgery (65 ± 8 minutes in the PRE group, 47 ± 7 minutes in the POST group and 50 ± 4 minutes in the control group) and time for extubation (4 ± 1 minutes in the PRE group, 3 ± 1 minutes in the POST group and 4 ± 1 minutes in the Control) were observed among groups. Mechanical ventilation was not necessary in any group. No clinical complications or adverse effects were observed during recovery from anesthesia in any group.

**Figure 1:**
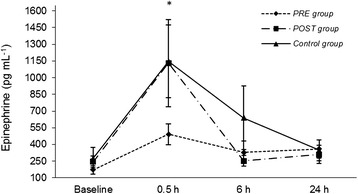
**Plasma epinephrine levels (mean ± SEM).** *PRE* dogs were treated with preemptive vedaprofen, *POST* dogs were treated with postoperative vedaprofen, *Control* dogs were treated with postoperative ketoprofen; *within a treatment, value differs significantly (P < 0.05) from the baseline value.

**Figure 2:**
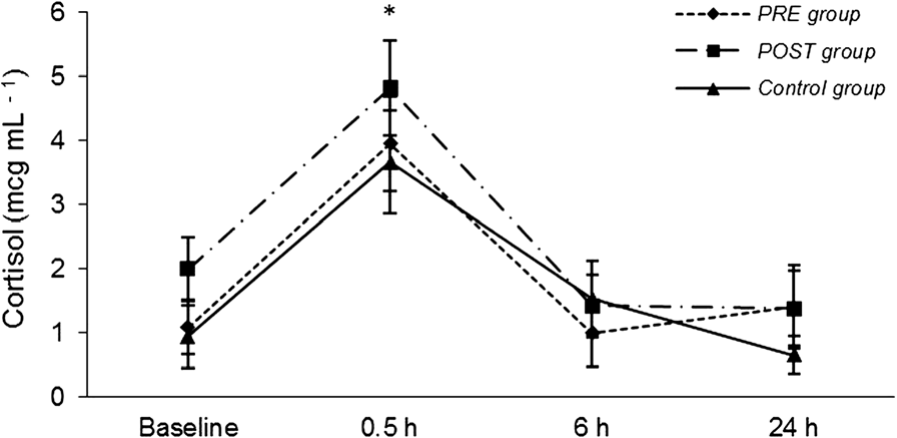
**Serum cortisol (mcg.mL** ^**−1**^ **) (mean ± SEM).** *PRE* dogs were treated with preemptive vedaprofen, *POST* dogs were treated with postoperative vedaprofen, *Control* dogs were treated with postoperative ketoprofen; *within a treatment, value differs significantly (P < 0.05) from the baseline value.

**Figure 3:**
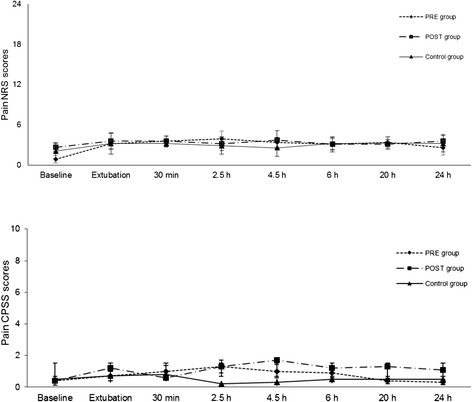
**Mean (SD) postoperative pain numerical rating scale (NRS).** *PRE* dogs were treated with preemptive vedaprofen, *POST* dogs were treated with postoperative vedaprofen, *Control* dogs were treated with postoperative ketoprofen.

**Figure 4:**
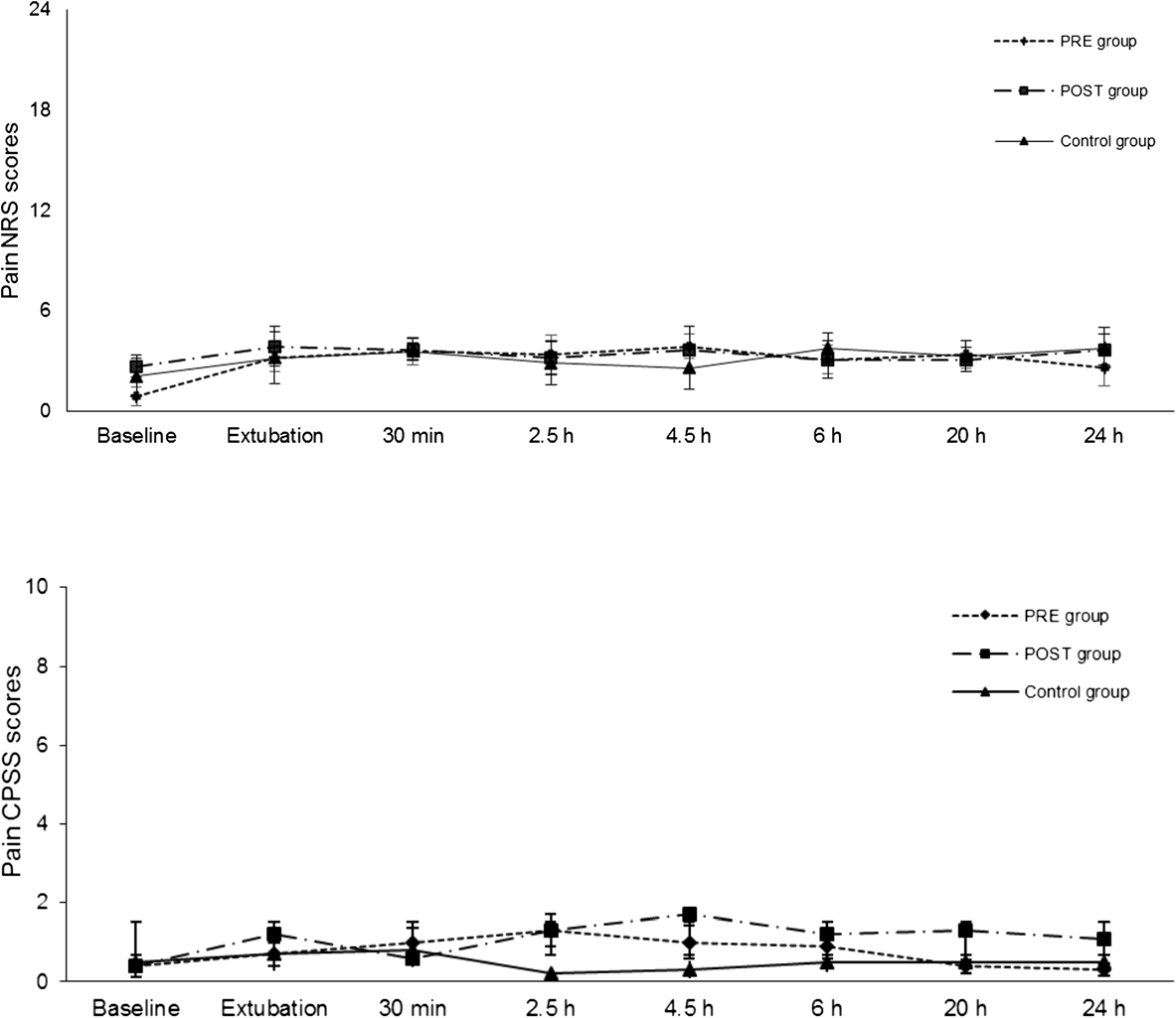
**Mean (SD) postoperative pain Colorado Pain Scoring System (CPSS).** *PRE* dogs were treated with preemptive vedaprofen, *POST* dogs were treated with postoperative vedaprofen, *Control* dogs were treated with postoperative ketoprofen.

In both groups, epinephrine and cortisol levels were significantly increased at 30 minutes after extubation compared to baseline (*P* < 0.05; Figures 1 and 2). Plasma norepinephrine and dopamine concentrations and pain and sedation scores were not significantly different within or among groups. There were no differences in analgesia or sedation scores within the groups or among them and rescue analgesia was not necessary in any dog (Figures 3 and 4). The highest scores in the NRS and CPSS were, respectively, 3.9 and 1.3 at 2.5 h in the PRE group; 3.7 and 1.7 at 4.5 h in the POST group and; 3.3 at 20 h and 0.8 at 30 minutes in the Control group.

Heart rate did not vary significantly throughout the study in any group. In the POST and control groups, the MAP (mean arterial pressure) was significantly decreased at extubation and at 30 minutes after extubation compared to baseline (*P* < 0.05). At 24 hours after extubation, the MAP was significantly increased (*P* < 0.05) and decreased (*P* < 0.05) compared with baseline in the POST and Control groups, respectively. The respiratory rate (f_R_) was significantly decreased at extubation and at 30 minutes, 2.5 h, 4.5 h and 6 h after extubation compared to baseline in all groups (*P* < 0.05). In the control group, the f_R_ was maintained significantly decreased at 20 h and 24 h after extubation compared with baseline and compared with PRE and POST groups at the corresponding time-points (*P* < 0.05). The rectal temperature was significantly decreased at extubation, which was maintained until 4 h (POST group) and 6 h (PRE and control groups) after extubation compared to baseline (*P* < 0.05). Heart rate, MAP, f_R_, and rectal temperature values were always within the range of normal values for the species (Table 1).

**Table 1:** **Vital parameters (mean ± SD)**

**Parameter**	**Group**	**Baseline**	**Extubation**	**Time after surgery**					
				**30 min**	**2.5 h**	**4.5 h**	**6 h**	**20 h**	**24 h**
HR	PRE	37 ± 8	129 ± 9	136 ± 12	139 ± 9	138 ± 10	136 ± 11	136 ± 11	125 ± 9
(beats.minute^−1^)	POST	138 ± 8	140 ± 12	137 ± 11	127 ± 9	128 ± 7	128 ± 7	124 ± 7	122 ± 6
	Control	122 ± 6	138 ± 9	141 ± 12	146 ± 6	127 ± 11	125 ± 10	136 ± 10	121 ± 10
f_R_	PRE	38 ± 7	24 ± 4*	18 ± 2*	27 ± 6*	21 ± 3*	20 ± 2*	36 ± 6	35 ± 6
(breaths.minute^−1^)	POST	43 ± 6	25 ± 4*	19 ± 1*	18 ± 1*	19 ± 2*	21 ± 3*	37 ± 7	36 ± 7
	Control	38 ± 5	24 ± 2*	21 ± 3*	17 ± 3*	15 ± 1*	21 ± 2*	25 ± 4*	18 ± 1*^†‡^
MAP	PRE	94 ± 7	83 ± 6	86 ± 6	85 ± 6	91 ± 8	94 ± 5	106 ± 4	91 ± 6
(mmHg)	POST	107 ± 5	87 ± 7*	95 ± 5*	98 ± 5	96 ± 4	109 ± 5	109 ± 5	114 ± 4†
	Control	105 ± 4	81 ± 3*	84 ± 5*	97 ± 6	94 ± 3	92 ± 3	96 ± 6	89 ± 6‡
Temperature	PRE	39.3 ± 0.2	37.1 ± 0.2*	36.9 ± 0.2*	37.5 ± 0.2*	37.8 ± 0.2*	38.1 ± 0.3*	39.2 ± 0.2	38.8 ± 0.2
(°C)	POST	39.2 ± 0.2	37.0 ± 0.3*	37.2 ± 0.3*	37.6 ± 0.3*	37.9 ± 0.2*	38.5 ± 0.2	39.2 ± 0.2	38.8 ± 0.2
	Control	38.9 ± 0.1	36.5 ± 0.3*	36.7 ± 0.2*	37.3 ± 0.3*	37.6 ± 0.2*	37.8 ± 0.2*	39.2 ± 0.2	39.1 ± 0.2

*HR* Heart rate, *f*_*R*_ respiratory rate, *MAP* mean arterial pressure, *PRE* dogs treated with preemptive vedaprofen, *POST* dogs treated with postoperative vedaprofen, *Control* dogs treated with postoperative ketoprofen; *within a treatment, value differs significantly (*P* < 0.05) from the baseline value; ^†^within a time-point, values are significantly different (*P* <0.05) from the PRE group; ^‡^within a time-point, values are significantly different (*P* <0.05) from the POST group.

## Discussion

The main finding of the present study was that the preoperative administration of sublingual vedaprofen was as effective as the postoperative administration for postoperative pain control, indicating that vedaprofen had no preventive analgesic effect in the present study. Regardless of the time of administration, vedaprofen given through the sublingual route was associated with low pain scores and was as effective as ketoprofen for controlling postoperative pain in dogs undergoing maxillectomy and mandibulectomy.

Vedaprofen was chosen in the present study based on pilot registers made up on the authors’ experience. The dosing was the one recommended by a pharmacokinetic study of vedaprofen in dogs, describing that absorption of vedaprofen is rapid and almost complete after oral administration, which is followed by a terminal half-live of the same order of magnitude of the intravenous administration [16]. In the present study, the advantages of using the sublingual route were that one did not have to force the animal to swallow the medication and the absorption of the drug was ensured even in dogs that did not have a swallow reflex (unconscious anesthetized). Vomiting was not observed in the present study, but the sublingual route would be another advantage over the oral route in these cases. The sublingual route was proven to be effective on absorbing vedaprofen, because both pre- and postoperative strategies resulted in pain scores as low as with ketoprofen, a NSAID known to be effective for controlling postoperative pain in dogs undergoing maxillectomy and mandibulectomy [19]. A group of dogs receiving ketoprofen was used as control due to ethical reasons, considering that the use of a placebo group is unfeasible in a study whose main objective is to find the best method to provide pain relief.

Among all analgesic agents used routinely, the NSAIDs are the less likely to block central sensitization resulting from surgery, since they primarily act peripherally by blocking the release of mediators that have been found to sensitize peripheral nociceptors, thus, leading to a reduction in inflammation and pain perception [20]. Even though, a previous study [6] found that dogs receiving carprofen, a NSAID, were awarded lower pain scores during the first postoperative hour than the group of dogs that received carprofen at the end of the surgery for a variety of orthopedic and soft tissue procedures. Therefore, one of the reasons for the lack of a preventive analgesic effect could be attributed to the fact that vedaprofen was administered in animals that were already experiencing some pain, considering the discomfort induced by the background disease that led to the indication for the surgical treatment. The crucial point in preventive analgesia strategies focuses in the administration of the analgesic before pain emerges, more than simply before surgical incision [3]. Accordingly, a previous study found a preventive analgesic effect of carprofen in dogs with no background painful disease that were anesthetized for elective ovariohysterectomy [7]. In another study, the authors did not find a preventive analgesic effect of carprofen in dogs with femoral or pelvic fractures, even though they had received the NSAID before surgical incision [21].

Alternatively, a preventive effect could have been revealed beyond the period covered by vedaprofen after a single administration (24 hours) [16]. This is because a study in human patients found that 86% of the patients who received preemptive epidural analgesia with opioids and local anesthetics were pain free at 9.5 weeks, in comparison with only 47% of the control patients [22]. This analgesic approach is more likely to block central sensitization because it is delivered directly into the epidural space. However, it was less likely to observe an improvement in pain control after long-term treatment with vedaprofen, considering that NSAIDs act reducing inflammation peripherally and, therefore, are expected to be more effective in the acute postoperative phase.

Another explanation for our findings could be attributed to the subjective judgment of pain scores. Since pain represents an unpleasant sensory and emotional experience, a direct measurement of a subjective experience is challenging and imposes difficulties, especially when evaluating it with a single tool. Therefore, in order to minimize variations, pain was assessed using two scoring systems in the present study. In addition, objective evaluations, such as physiological data, and serum cortisol, epinephrine, norepinephrine and dopamine were measured as indirect measurements of pain, in order to reduce the subjective nature of the pain scores. The stress caused by a painful stimulus can increase heart rate, MAP, f_R_ and serum cortisol and catecholamine levels [23]. Therefore, it is unlikely that the postoperative decrease in f_R_ and temperature compared to baseline was a response to pain. In fact, these changes were found in all groups, suggesting they were attributed to the residual effects of isoflurane, rather than caused by differences in the NSAID used or in the time they were administered [24]. Less likely explanations could be the residual effects of acepromazine or an increased f_R_ and temperature at baseline caused by fear and stress experienced prior to the pre-anesthetic medication [25].

The preoperative vedaprofen induced no significantly differences in the serum cortisol and catecholamine levels compared to the postoperative administration. In both groups, the norepinephrine and dopamine concentrations did not vary significantly throughout the study, which is in accordance with the other measurements. Nevertheless, both groups had increased cortisol and epinephrine levels at 30 minutes post-extubation compared to baseline. This was the only pain indicative in the present study and it was not in-line with the changes observed in pain scores and physiological variables. The cortisol levels, however, were similar to a previous study that found an effective pain control in dogs receiving tramadol, codeine, ketoprofen alone or in combination for the same type of oral orthopedic surgical procedures [19]. Therefore, the changes on cortisol and epinephrine were attributed to a stress response that probably was not caused by pain. For instance, the stress caused by a different environment (e.g. veterinary hospital, cage) is capable of activating the pituitary-adrenal system in dogs [23]. Despite of the hormonal postoperative changes, the dogs had no other signs of pain. They all had good appetite and ate normally when they were offered food 5 hours after surgery, which supports the fact that the increase in cortisol and epinephrine levels was due to stress not induced by pain.

If the analgesic efficacy for the pre- over the postoperative administration was not proven advantageous, then the lack of information about the potential intraoperative adverse effects could preclude their preoperative use [26]. One may choose to start the analgesic therapy with NSAIDs at the end of surgery in order to prevent a possible increase in bleeding time intra-operatively [24,27]. However, since vedaprofen is known to be a preferential cyclo-oxygenase-2 inhibitor in dogs [18], it would be advantageous for causing less adverse effects than other conventional NSAIDs, allowing long-term treatment with low incidence of gastrointestinal and renal adverse effects or on increase in bleeding time [28]. However, controversial findings were reported on clinical studies in dogs. A multicenter clinical trial reported that mild and transient side effects, such as diarrhea, vomiting and loss of appetite, were observed in 11% and severe side effects, such as bloody diarrhea and/or repeated vomiting, were observed in 5% of dogs with musculoskeletal disorders receiving vedaprofen for 14 days [14]. Another study described mild and transient side effects in 17.1% of the dogs and treatment had to be stopped in 6.7% of the dogs receiving vedaprofen for 56 days [13]. The incidence of these adverse effects was similar to that found with the meloxicam in both studies. In addition, in other species, such as horses, vedaprofen was found to be cyclo-oxygenase-1 selective in experimental studies [29], although clinical studies in this species showed no such adverse effects [30]. In the present study, although data were not recorded during long-term treatment, adverse effects were not observed in any dog during surgery or within 24 hours of administration of vedaprofen.

Although the preventive analgesia concept supports the assumption that blocking central sensitization reduces overall pain perception and duration, it is not known, however, at which point is preventive intervention most important, if it is blocking the nociceptive input at surgery or blocking the postoperative pain resulting from surgery in the immediate postoperative period. A study in humans indicates that whether or not the nociceptive input of surgery was blocked through the administration of long acting local anesthesia, overall postoperative pain perception was the same [31]. Therefore, regardless of the timing of the vedaprofen administration, both pre- and postoperative strategies are ethically justified by their efficacy on pain relieve observed in the present study.

## Conclusions

In conclusion, the results of the present study showed that vedaprofen had no preventive analgesic effect, since the preoperative administration had no advantages over the postoperative administration. However, both pre- and postoperative vedaprofen given by the sublingual route were as effective as ketoprofen administered by the intramuscular route for postoperative pain control in dogs undergoing mandibulectomy and maxillectomy.

## Methods

### Animals

The study was approved by the Comissão de Ética no Uso de Animais (CEUA; Ethics Committee on Animal Use) of Faculdade de Medicina Veterinária e Zootecnia from Universidade de São Paulo, under the protocol #1667260314. Thirty client-owned dogs undergoing maxillectomy or mandibulectomy for oral neoplasms removal were evaluated. The minimum sample size for paired data was calculated using power analysis and 10 dogs per group were required to have 95% chance (with 5% risk) to detect a difference of 2.5 mm in the pain score between groups and considering a standard deviation of 4 mm in the population. The study population of the PRE group consisted of 5 males and 5 females, comprising 4 mixed-breed dogs, 3 Poodles, 2 Cocker Spaniels and 1 Maltese. In the POST group, there were 6 males and 4 females, which comprised 4 Poodles, 3 Cocker Spaniels, 2 mixed-breed dogs and 1 Airdale. In the Control group, there were 6 males and 4 females, which were 4 Poodles, 3 Cocker Spaniels, 2 mixed-breed dogs and 1 Bichon Frise. Animals were only included in the study if they had mandible or maxillary neoplasm confirmed by clinical examination and other evaluations, such as radiography, ultrasonography, tomography, cytology or histopathology; if they were scheduled for maxillectomy or mandibulectomy surgery in the morning period and; if no clinical cardiopulmonary, renal or hepatic abnormalities were detected after a complete physical examination, blood cell count and serum biochemical analysis were performed. Animals were excluded from the study if they had a contraindication for NSAIDs (i.e. clinical signs of gastrointestinal, hepatic or renal disease). Client-consent was obtained before entry of any dog into the study.

### Anesthesia and surgery

Food was withheld for 12 hours and water for 4 hours before anesthesia. All dogs received acepromazine (0.05 mg.kg^−1^ IM; Acepran-Univet, São Paulo, Brazil) as a pre-anesthetic medication and after 10 minutes, a 20-gauge catheter was fixed in the left cephalic vein for intravenous (IV) administration of drugs and fluids. Anesthesia was induced with propofol (5 mg.kg^−1^; Propovan, Cristalia, Itapira, Brazil) administered slowly through the IV catheter. After orotracheal intubation, anesthesia was maintained with an end-tidal isoflurane concentration of 1.6-1.8% (Isoforine, Cristalia, Brazil) vaporized in 100% oxygen via a semi-closed rebreathing circuit. A side-stream gas analyzer (Multinex Plus, Datascope, New York, USA) was connected between the endotracheal tube and the Y-piece to measure the end-tidal isoflurane concentration, end-tidal carbon dioxide tension (ETCO_2_) and the f_R_. Animals were allowed to breath spontaneously, unless ETCO_2_ was not adequate (35–45 mmHg), in which case mechanical ventilation could be used. The need for mechanical ventilation, if any, was registered. Heart rate and rhythm, and NIBP were monitored with a multiparametric data collection system (Viridia-M3, Hewllet Packard, Germany). Fluid therapy with lactated Ringer’s solution was infused at 10 mL.kg ^−1^.hour^−1^ during anesthesia. An 18-gauge catheter was placed in the jugular vein of each animal to facilitate blood sampling.

The surgical procedure was always performed by the same senior surgeon, using the same resection pattern and suture material for each surgery. Following surgery, the dogs were extubated after laryngeal reflexes were restored and then allowed to recover from anesthesia on a heated mat in a cage. Duration of surgery and the time between the disconnection of the orotracheal tube from the anesthetic system and extubation were recorded. All dogs were offered water *ad libitum* after recovery from anesthesia. Soft or liquefied food was offered during the period from 5 to 12 hours after surgery.

### Study design

Animals randomly received one of the following treatments:

- Vedaprofen (0.5 mg.kg^−1^, sublingual; Quadrisol, Boxmeer, Netherlands) at the time of premedication (30 minutes before surgery) and saline at 10 minutes prior to the end of surgery (PRE group; *n* = 10).
- Saline at the time of premedication (30 minutes before surgery) and vedaprofen (0.5 mg.kg^−1^, sublingual; Quadrisol, Boxmeer, Netherlands) at 10 minutes prior to the end of surgery (POST group; *n* = 10)
- Saline at the time of premedication (30 minutes before surgery) and ketoprofen (2 mg.kg^−1^ IM; Ketofen, Rhodia-Merieux, Paulinia, Brazil) at 10 minutes prior to the end of surgery (Control group; *n* = 10).

The reason to administer the drugs at 10 minutes before the end of the surgery was to ensure that they would have enough time to be absorbed and that they would have reached the adequate plasma concentration by the time surgery was finished.

Randomization was performed using sealed numbered envelopes. Drug preparation and administration, and evaluations were blind.

### Postoperative assessment

Analgesia was assessed using two scales: a 10-category numerical rating scale (NRS; see Additional file 1) [28], in which 0 corresponds to no pain and 10 to a patient in extreme pain; and the Colorado Pain Scoring System (CPSS; see Additional file 2) [32], which ranges from 0 (no pain) to 4 (extreme pain). Pain was always scored by the same blind observer during the study. Animals could be rescued with morphine (0.1 mg.kg^−1^ IM) if NRS scores were ≥4 and CPSS scores were ≥2. The total number of rescue analgesic, if any, was recorded.

Adverse effects, such as vomiting, diarrhea and sedation were recorded throughout a 24-hour observation period after surgery. A sedation scale that ranged from 0 to 3 was used, at which 0 = no sedation, no sensory or motor deficits; 1 = mild sedation, slight sensory or motor deficits evident as ataxia or disorientation; 2 = moderate sedation, sternal recumbency and slight protrusion of the third eyelid; and 3 = strong sedation, lateral recumbency and accentuated protrusion of the third eyelid [33].

Heart rate, MAP (oscillometric method), f_R_ and rectal temperature were recorded, in addition to the pain and sedation scores, prior to premedication (baseline), immediately after extubation and at 0.5 (30 minutes), 2.5, 4.5, 6, 20 and 24 hours after extubation. Dogs included in the study were treated for as long as necessary, but data for the study were recorded only up to 24 hours after surgery.

### Laboratory data

These data were measured in duplicate in the laboratory of the Faculty and were performed at baseline, and at 30 min, 6 h and 24 h after extubation. Venous blood samples of 1.5 mL were collected from the jugular catheter to measure cortisol and catecholamines (epinephrine, norepinephrine and dopamine) levels. Samples to measure cortisol were withdrawn in plastic syringes and transferred to glass tubes, which were then centrifuged at 1500 rpm for 10 minutes and frozen at −20°C for later analysis using commercial immunoassay kits (Autodelfia Cortisol Kit, Wallac, Finland). The detection limit for cortisol was 15 nmol.L^−1^, with a 1.4% interassay coefficient of variation and a 3.1% intra-assay coefficient of variation. Blood samples to measure catecholamines were withdrawn using a specific anticoagulant (glutathion + ethylene glycol tetra acetic acid) and then immediately centrifuged at 3000 rpm at 4°C for 10 minutes in a cold centrifuge (Model RT7, Sorvall, California, USA). Resulting serum were then stored at −80°C until analysis using high-performance liquid chromatography (Model 460, Waters, São Paulo, Brazil), according to the methodology described by Krstulovic [34]. The intra-assay coefficients of variation for the measurement of catecholamines in venous plasma were 5.5% for both epinephrine and norepinephrine, and the inter-assay coefficients were 6.8% for norepinephrine and 7.9% for epinephrine. The limits of detection were 12.5 pg.mL^−1^ for both epinephrine and norepinephrine.

### Statistical analysis

The physiologic data were analyzed by parametric examination within groups and between groups by using analysis of variance (ANOVA) for repeated measurements. When appropriate, a *post hoc* analysis was performed with the Tukey test. For nonparametric data, such as NRS, CPSS and sedation scores, Friedman and Kruskal-Wallis tests were employed followed by *post hoc* Dunn’s test. The results were considered significant if *P <*0.05.

## Additional files

Additional file 1:
**10-category Numerical Rating Scale.**
Additional file 2:
**Colorado State University Canine Acute Pain Scale.**
