# Supplementary material for: A comparison of pre and post-operative vedaprofen with ketoprofen for pain control in dogs
Source: BMC Vet Res. 2015 Feb 7;11:24. doi: 10.1186/s12917-015-0338-4 (PMC4324799; doi:10.1186/s12917-015-0338-4)
Supplement: Additional file 1: — 10-category Numerical Rating Scale. [file 12917_2015_338_MOESM1_ESM.pdf]

Appendix 1 Numerical rating scale used for scoring postoperative pain in dogs

Score	Criteria
0	No pain. Patient is running, playing, eating. Sleeping comfortably with dreaming. Heart rate should be normal, but if elevated, it is due to excitement.
1	Probably no pain. Patient appears to be normal but condition is not as clear-cut as above. Heart rate should be normal, or slightly increased due to excitement.
2	Mild discomfort. Patient will still ear or sleep but may not dream. May limp slightly or resist palpation of the surgical wound, but otherwise shows no other signs of discomfort. Not depressed. There may be a slight increase in respiratory rate; heart rate may or may not be increased.
3	Mild pain or discomfort. Patient will limp or guard incision and looks a little depressed. Cannot get comfortable. Appears to be interested in food and may still eat a little but somewhat picky. Respiratory rate may be increased and a little shallow.
4	Mild to moderate pain with the patient resisting touching of the operative site. May or may not appear interested in food. Respiratory rate may be increased or shallow.
5	Moderate pain. Condition progressing from above or patient reluctant to move, depressed, inappetant and may bite or attempt to bite when the caregiver approaches the painful area. The patient may vocalize when caregiver attempts to move the patient or when it is approached. The heart and respiratory rates may be increased. The patient is not interested in food, will lie down but does not really sleep.
6	As above, but patient may vocalize or whine frequently, without provocation and when attempting to move. Heart rate will be increased.
7	Moderate to severe pain. The patient is very depressed and is not concerned with its surroundings. The patient will urinate and defecate without attempting to move

	and will cry out with slight movement, or will spontaneously or continually whimper. Heart and respiratory rates are increased.
8	Severe pain. Signs as above (7). Vocalizing may be more of a feature, or being so consumed with pain, the patient will not notice your presence. The patient may thrash around the cage intermittently.
9	Severe to excruciating. As above (8), but patient is hyperesthetic. The patient will tremble involuntarily when any part of the body is close proximity to wound or injury. Tachycardia may still persist and it may be impossible to control the pain. This degree of pain can cause death.
10	As above (9), but patient emitting piercing screams or almost comatose. The patient is hyperesthetic/hyperalgesic. The whole body is trembling, and pain is elicited wherever you touch the patient. This degree of pain can cause death.
